# A Culinary and Medicinal Gem: Exploring the Phytochemical and Functional Properties of Thai Basil

**DOI:** 10.3390/foods13040632

**Published:** 2024-02-19

**Authors:** Panita Prasongdee, Kakanang Posridee, Anant Oonsivilai, Ratchadaporn Oonsivilai

**Affiliations:** 1 School of Food Technology, Institute of Agricultural Technology, Suranaree University of Technology, Nakhon Ratchasima 30000, Thailand; p.prasongdee@gmail.com (P.P.); posridee.ka@gmail.com (K.P.); 2 School of Electrical Engineering, Institute of Engineering, Suranaree University of Technology, Nakhon Ratchasima 30000, Thailand; 3 Health and Wellness Research Unit, Suranaree University of Technology, Nakhon Ratchasima 30000, Thailand

**Keywords:** Thai basil, phenolic, flavonoid, antioxidant activity, DCFH-DA

## Abstract

This study aimed to comprehensively evaluate the potential health benefits of Thai basil extracts from two species, *Ocimum basilicum* var. *thyrsiflorum* and *Ocimum basilicum* cv. Jumbo 4320, by investigating their bioactivities, phytochemical composition, and in vitro antioxidant, antimicrobial, and antithrombotic activities. Thai basil extracts from two species (*Ocimum basilicum* var. *thyrsiflorum* and cv. Jumbo 4320) were obtained using water, ethanol, and ethyl acetate. Phytochemical analysis revealed chlorophylls, carotenoids, and diverse phenolic compounds. Its water extract boasted the highest total phenolics (459.62 ± 3.07 mg GAE/100 g), outperforming ethanol and ethyl acetate extracts (171.20 ± 1.10 and 66.02 ± 0.99 mg GAE/100 g, respectively). The ethanol extract of Jumbo 4320 also reigned supreme in total flavonoids (557.12 ± 14.27 mg CE/100 g), surpassing its counterparts (209.07 ± 23.31 and 131.41 ± 0.37 mg CE/100 g). *O. basilicum* cv. Jumbo 4320 extracts exhibited superior antioxidant activity by various assays. Jumbo 4320′s water extract further dominated in the DPPH assay (IC_50_ 48.52 ± 1.15 mg/mL), demonstrating superior free radical scavenging compared to ethanol and ethyl acetate extracts (IC_50_ 60.54 ± 0.52 and 82.09 ± 3.18 mg/mL), respectively. While *thyrsiflorum*’s ethanol extract claimed the top spot in the FRAP assay (0.0186 ± 0.00 mmol Fe^2+^/g), Jumbo 4320′s water extract showcased the highest cellular antioxidant activity (80.62 ± 0.00% relative fluorescence intensity) in the DCFH-DA assay. Interestingly, *Ocimum basilicum* var. *thyrsiflorum* extracts demonstrated stronger antithrombotic activity at prolonging the prothrombin time at 78.3 ± 17.56 s. While the antimicrobial activity against most tested pathogens was limited, both species’ extracts inhibited Bacillus cereus. These findings suggest the potential of Thai basil extracts, particularly from Jumbo 4320, as functional food ingredients with antioxidant and antithrombotic properties.

## 1. Introduction

*Ocimum basilicum* Linn. (*O. basilicum*), commonly known as sweet basil, boasts a rich culinary history and traditional medicinal uses. In recent years, scientific research has delved deeper into its bioactive potential, uncovering a wealth of health benefits. Basil extracts have consistently demonstrated potent antioxidant properties due to their abundance of phenolic compounds and flavonoids. Studies by Chang et al. [1] and Luo et al. [2] showcase their protective effects against oxidative stress in HepG2 cells, while Kim et al. [3] and Choi et al. [4] highlight the impact of cultivar and solvent choice on antioxidant capacity. Novel research into previously unreported bioactive components offers new avenues for understanding and optimizing basil’s antioxidant potential [3,4]. The antibacterial properties of *O. basilicum* extracts have attracted significant attention due to the rising concern of antibiotic resistance. Investigations by Benković et al. [5] and Burt et al. [6] established broad-spectrum activity against various bacterial strains, including multidrug-resistant pathogens. This research on targeted extraction methods for enhanced antibacterial activity against specific bacteria contributes valuable insights to this field.

The potential of *O. basilicum* extracts to prevent blood clotting has emerged as a promising area of investigation. Gadag et al. [7] highlight the role of prothrombin time assays in assessing antithrombotic activity, while this research sheds light on the specific compounds and extraction techniques responsible for this effect. Further exploration of mechanisms of action and in vivo studies are crucial for advancing this research. Beyond these key areas, *O. basilicum* extracts have shown potential in alleviating inflammatory disease, protecting against neurodegenerative conditions, and even exhibiting antidiabetic properties [2,8,9].

The past five years have witnessed significant advancements in understanding the health benefits and therapeutic potential of *O. basilicum* extracts. From potent antioxidant and antibacterial properties to promising antithrombotic activity and diverse other health benefits, the research underscores the remarkable potential of this humble herb. By addressing existing challenges and embracing innovative approaches, like in this research, the future of *O. basilicum* extract research is ripe with possibilities for advancing human health and well-being.

While *Ocimum basilicum* (sweet basil) extract is not currently approved for medical use, early research suggests intriguing potential health benefits. The extract exhibits antimicrobial activity against bacteria, potentially aiding food preservation and infection control [5,6]. It also possesses antioxidant and neuroprotective properties, suggesting benefits for chronic diseases and neurodegenerative disorders like Alzheimer’s [7,8]. Preliminary research even hints at antithrombotic activity [7]. However, these findings are based on preclinical studies, and further clinical trials are crucial to confirm the safety and efficacy in humans. Additionally, determining optimal dosage, formulation, and potential drug interactions requires dedicated research before therapeutic applications can be considered. Remember, responsible reporting necessitates accurately representing the current state of knowledge and avoiding premature claims about pharmaceutical applications. This extract’s future holds promise, but extensive research is necessary before it can be considered a safe and effective medical treatment [5,6,7,8,9].

Asian traditional medicine (ATM) offers a rich tapestry of natural ingredients with potent antioxidant properties, holding immense potential for enhancing our well-being. This review delves into the diverse sources of these antioxidants, including plants like ginseng, turmeric, green tea, and ginger, each brimming with bioactive compounds like phenolic acids and flavonoids. These compounds act as shields, scavenging free radicals, reducing oxidative stress, and modulating inflammation.

The applications of ATM antioxidants in food are vast and exciting. We can directly incorporate these ingredients into functional foods, boosting their nutritional value and functionality, like adding green tea extracts to beverages or turmeric to spices and curries. Their antioxidant power can also extend shelf life and reduce spoilage in food products, as seen with rosemary extracts in oils or ginger in marinades. Additionally, specific bioactive compounds, like curcumin from turmeric, can be isolated for targeted applications in novel functional foods.

Examples abound: green tea, a well-known source of polyphenols, finds its way into beverages, desserts, and even supplements. Turmeric, with its potent curcumin, adds flavor and health benefits to spices, curries, and functional drinks. Ginseng, an adaptogenic herb, offers antioxidant and immune-modulating properties when enjoyed in teas, soups, and energy drinks. Goji berries, rich in lycopene and zeaxanthin, can be consumed raw, dried, or incorporated into juices and yogurts.

However, challenges remain. Ensuring the consistent quality and potency of ATM ingredients is crucial, as is consumer acceptance through careful taste and sensory considerations. Navigating food safety regulations and legal frameworks for using traditional medicine in food products also requires attention.

Looking ahead, research needs to delve deeper into the specific mechanisms of action, synergistic effects, and optimal bioavailability of ATM antioxidants in food applications. Innovation in food formulations and delivery systems will further unlock their potential. Finally, promoting sustainable cultivation and ethical sourcing practices for ATM plants used in food products is essential.

This study aimed to comprehensively evaluate the potential health benefits of Thai basil extracts from two species, *Ocimum basilicum* var. *thyrsiflorum* and *Ocimum basilicum* cv. Jumbo 4320, by investigating their bioactivities, phytochemical compositions, and in vitro antioxidant, antimicrobial, and antithrombotic activities.

## 2. Materials and Methods

### 2.1. Plant Material

Thai basil leaves of two varieties, thyrsiflorum and Jumbo 4320, were meticulously prepared in Nakhon Ratchasima, Thailand. After careful rinsing, they were dried at 50 °C for a day, transformed into fine powder using an Ultra Centrifugal Mill Model ZM-1000 (Retch, Haan, Germany), sieved by mesh with a size of 0.2 mm, and stored in airtight vacuum packs at −20 °C for future use. This meticulous process ensures the highest quality and preservation of the basil’s potent properties.

### 2.2. Chemical and Standards

Chemicals, reagents, and standards were meticulously sourced by Sigma-Aldrich Co. (St. Louis, MO, USA), Difco (Detroit, MI, USA), and Mallinckrodt-Baker (Phillipsburg, NJ, USA), and prepared, ensuring quality and reproducibility for the following experiments. HPLC-grade solvents and standards were employed for precise analysis, while pheophytins were synthesized from chlorophylls following established protocols. This thorough groundwork lays the foundation for reliable data acquisition in the upcoming research.

### 2.3. Proximate Analysis

Dry Thai basil powders were analyzed for their chemical composition. Moisture analysis was performed by AOAC method 925.10. Ash and sand quantification were performed using AOAC method 900.02A and 900.02D, respectively. Protein and fat quantification were conducted by AOAC method 928.08 and 963.15, respectively. Crude fiber determination was performed by AOAC method 978.10. (AOAC, 2005) [10].

### 2.4. Preparation of Thai Basil Extracts

About 5.0 g of Thai basil powder was extracted with 50 mL portions of boiling water, ethanol, and ethyl acetate by shaking for 18 h at a temperature of 37 °C, and then centrifuged at 2800 rpm (Thermo Scientific, Braunschweig, Germany) for 15 min; then, they were filtered and the volumes were adjusted to 50 mL with the same solvent. Two milliliters of each extract were then transferred to test tubes. Solvent removal was achieved via rotary evaporation for ethanol and ethyl acetate, and freeze-drying for the water extract. All samples were stored at −20 °C until needed (adapted from [11]). Quantification of the extracts assumed the complete extraction of the active ingredients from the raw materials, which was applied throughout the experiments [11].

### 2.5. Phytochemical Profiling

#### 2.5.1. Chlorophyll and Carotenoid Profiling of Thai Basil Extracts

The HPLC was employed to quantify the chlorophyll and carotenoid content in Thai basil extracts, based on the method of Oonsivilai et al. [12]. Briefly, separation was determined using a Grace-Vydac 201TP54 reversed-phase (4.6 mm × 250 mm) polymeric C18 column with a guard column containing the same stationary phase (Grace Vydac, Apple Valley, MN, USA). A gradient elution profile was used based on a binary mobile phase system consisting of methanol:water: ammonium acetate (73:25:2, *v*/*v*/*v*) in reservoir A and ethyl acetate in reservoir B. A flow rate of 1.0 mL/min was applied with an initial setting at 100% (A) and with a linear gradient to 50:50 (A/B) over 10 min. The gradient was held for 10 min, followed by a 5 min linear gradient back to 100% (A), and equilibration at the initial condition for 5 min, for a total run time of 30 min. Chlorophyll a and b were identified by comparing retention times and spectra with standards at 250–600 nm. Quantification was based on calibration curves with linear ranges of 0.1–5 ppm for both chlorophylls (correlation coefficients > 0.99).

#### 2.5.2. Phenolic Profiling of Thai Basil Extracts

An adapted Oonsivilai et al. [12] method employed HPLC-quantified phenolic compounds in the Thai basil extracts. Separation was achieved on a Waters C18 column at 35 °C with a 1.0 mL/min flow rate. A binary mobile phase gradient elution was used, with water/acetic acid (98:2) in reservoir A and acetonitrile in reservoir B. Starting at 99:1 A/B, the elution transitioned linearly to 70:30 A/B over 20 min, was held for 5 min, then returned to 99:1 A/B over 5 min, followed by 5 min of equilibration. In-line PDA data between 200 and 500 nm facilitated the detection and tentative identification of phenolic compounds. Quantification relied on calibration curves constructed from known standards of gallic acid, catechin, caffeic acid, coumaric acid, ferulic acid, sinapic acid, and apigenin, ranging from 5 to 50 ppm.

### 2.6. Total Phenolic Contents

The amount of total soluble phenolics in the extracts was measured using a method by Singleton et al. [13] and a chemical called Folin–Ciocalteu reagent. An aliquot (20 µL) of the samples was added to a test tube; after that, 1.58 mL of DI water was added, followed by 100 µL of Folin–Ciocalteu reagent; then, the mixture was thoroughly mixed and further incubated for 5 min at room temperature. Following incubation, 300 µL of the Na_2_CO_3_ (20% *w*/*v*) solution was added and the mixture was allowed to stand at room temperature in the dark for 2 h. Absorbance was measured at 765 nm using a spectrophotometer (Biochrom Libra S22, Cambridge, UK). Gallic acid (50, 100, 250, 500, and 750 ppm (mg/L)) was used as the standard. Results were expressed as gallic acid equivalents.

### 2.7. Total Flavonoid Contents

The method of Qu et al. [14] with aluminum chloride was applied and catechin as a reference was used to quantify the total flavonoids in the extracts. An aliquot (250 µL) of the samples was added to a test tube. After that, 1.25 mL of DI water was added, followed by 75 µL of 5% NaNO_2_; then, the mixture was incubated for 6 min. Following incubation, 150 µL of the 10% AlCl_3_ solution was added, followed by 0.5 mL of 1 M NaOH and 275 µL of DI water, respectively. The mixture was thoroughly mixed and incubated for 5 min. Absorbance was measured at 510 nm using a spectrophotometer (Biochrom Libra S22, Cambridge, UK). Catechin (0–350 ppm (mg/L)) was used as the standard. Results were expressed as catechin equivalents.

### 2.8. DPPH Radical Scavenging Activity

The free radical scavenging activity of Thai basil extracts (water, ethanol, and ethyl acetate) was assessed using the DPPH assay, following the method of Oonsivilai et al. [12,15]. BHT and ascorbic acid in methanol served as the positive controls. Inhibition of free radical DPPH was calculated according to the formula:

$$\%\;\mathrm{Inhibition}=\left[\frac{\left(Ab-As\right)}{Ab}\right]\times 100$$

where Ab is the absorbance of sample Blank; As is the absorbance of the sample; from a plot of concentration against % inhibition, a linear regression analysis was performed to determine the IC_50_ or extract the concentration, resulting in a 50% inhibition value for each sample. The IC50 values of the extracts were calculated in Sigma Plot 9.1.

### 2.9. Ferric-Reducing Antioxidant Power Assay

Following the method described by Samruan et al. [16], the ferric-reducing antioxidant power (FRAP) assay was employed to assess the antioxidant capacity of the extract. Briefly, the working FRAP reagent was prepared by acetate buffer (pH of 3.6), a 10 mmol TPTZ solution in 40 mmol HCl, and a 20 mmol ferrous chloride solution in the proportion of 10:1:1 (*v*/*v*). Freshly prepared FRAP reagent was warmed to 37 °C in a water bath prior to use. The extract of 50 µL was added to 1.5 mL of the FRAP reagent and incubated for 4 min. Absorbance was measured at 593 nm using a spectrophotometer (Biochrom Libra S22, Cambridge, UK). All solutions were used on the day of preparation. The BHT was used as positive controls and the standard curve was prepared using different concentrations of the ferric sulphate solution (0.2–1 mM). The results were expressed as µmol equivalents of ferric per g dry weight of the plant materials.

### 2.10. Cell Culture

This research employed the HepG2 cell line, derived from human liver cancer tissue (ATCC catalog number HB-8065). These cells were cultured in a specific medium called DMEM, enriched with 10% fetal bovine serum, 2 mM L-glutamine, and 1% penicillin-streptomycin solution. The cell culture was maintained at an optimal temperature of 37 °C within a fully humidified incubator, providing a controlled atmosphere of 5% carbon dioxide and 95% air.

### 2.11. Cytotoxicity of Extracts

Following established protocols, 0.5 × 10^4^ cells/well human liver cancer cells were seeded in a 96-well plate and incubated for 24 h under controlled conditions (37 °C, 5% CO_2_ in 95% air). Subsequently, Thai basil extracts, dissolved and diluted in a culture medium containing 0.1% DMSO (Amresco, Solon, OH, USA), were added at various concentrations and incubated for another 24 h. After the incubation period, the extracts were removed and the cells were washed with PBS (Gibco, Gaithersburg, MD, USA) to prepare them for subsequent analysis.

Cell viability was determined using the MTT assay. One hundred microliters of culture medium containing MTT (a metabolic activity indicator) (Invitrogen, Carlsbad, CA, USA) was added to each well and incubated for 4 h. The MTT solution was then removed, and the cells were washed with PBS. DMSO, a solvent, was added to dissolve the purple formazan crystals produced by viable cells. Finally, the absorbance at 570 nm was measured using a microplate reader (Bio-Rad Benmark Plus, CA, USA), providing an optical quantification of viable cells based on their metabolic activity.

The experiment included two control groups: a reagent control containing only culture medium and a solvent control containing 0.1% DMSO, the solvent used to dissolve the extract. A dose–response curve was generated using five different concentrations of the extract ranging from 50 to 500 μg/mL. The results were expressed as the lethal concentration 50% (LC50), which represents the concentration of the extract required to reduce cell viability by 50% compared to the control groups. This value provides an indicator of the extract’s cytotoxicity, or its ability to harm living cells.

### 2.12. Cellular Antioxidant Activity (CAA) by DCFH-DA Assay

We investigated the ability of Thai basil extracts to protect cells from oxidative damage (cellular antioxidant activity) using a modified protocol based on a previous study by Wolfe et al. [17]. The experiment used human liver cancer cells (HepG2) cultured in a 96-well plate. Each well was seeded with 60,000 cells (6 × 10^4^) and incubated with 100 microliters of growth medium for 24 h. After incubation, the growth medium was discarded, and the cells were gently washed with a buffer solution (PBS) to remove any remaining medium. Each well then received 100 microliters of a Thai basil extract solution and incubated for another hour to allow the extract to interact with the cells. After the treatment, the extract solution was removed and the cells were washed again with PBS to remove any residual extract. Next, a chemical called 2′,7′-dichlorofluorescein diacetate (DCFH-DA) was added to each well and incubated for 30 min. This chemical becomes fluorescent when it reacts with reactive oxygen species (ROS) inside the cells, allowing us to measure the level of oxidative stress. Depending on the specific experiment being conducted, an optional washing step with PBS might have been performed after the DCFH-DA incubation. Finally, to trigger the production of ROS inside the cells, a different chemical called 2,2′-azobis[2-amidinopropane] hydrochloride (ABAP) was added to each well in a solution called Hank’s balanced salt solution (HBSS). By measuring the fluorescence intensity of DCFH-DA after the ABAP treatment, the effectiveness of the different Thai basil extracts could be compared in protecting cells from oxidative damage. A fluorescence intensity at 538 nm (excitation at 485 nm) was monitored every 5 min for 1 h using a Spectramax Gemini EM fluorescence microplate reader (Molecular Devices, Sunnyvale, CA, USA). Each plate included control wells with cells treated with DCFH-DA and ABAP (oxidative stress control), and blank wells containing cells treated only with DCFH-DA and HBSS (basal fluorescence control). Vitamin E served as a positive control for antioxidant activity.

### 2.13. Antimicrobial Activity

#### 2.13.1. Microbial Strains

The antimicrobial activity of the samples was assessed against a diverse panel of six bacterial strains: *Bacillus cereus* (TISTR687), *Bacillus subtilis* (TISTR008), *Enterobacter aerogenes* (bcc6719), *Escherichia coli* (TISTR3436), *Pseudomonas aeruginosa* (TISTR781), and *Staphylococcus aureus* (TISTR1466). Before testing, each bacterial strain was cultivated overnight at 37 °C in Mueller-Hinton agar (MHA).

#### 2.13.2. Disk Diffusion Method

We delved into the antimicrobial potential of the extracts using the well-established agar disk diffusion method [18]. This meticulous process began with the overnight incubation of broth cultures to ensure ample bacterial density. Turbidity was then adjusted to a standardized 10^8^ colony-forming units (CFU)/mL, meticulously controlled by either a McFarland nephelometer or visual comparison to the 0.5 McFarland standard. A consistent bacterial “lawn” was established by spreading 100 µL of the adjusted suspension onto Mueller–Hinton agar plates. Tiny 6 mm filter paper disks, imbued with 10 µL of the extract solutions, were strategically placed on the plates. To ensure accurate interpretation, each plate included two control disks: one containing 75% DMSO in the solvent as a negative control and another infused with chloramphenicol (Sigma Aldrich, St. Louis, MO, USA), a known antibiotic, as a positive benchmark. Following incubation at 37 °C for 24 h, we meticulously measured the diameter of any clear zones surrounding the disks, revealing the extracts’ ability to inhibit bacterial growth compared to the controls. Each sample was tested in triplicate, with reported values representing the average of these three measurements. Through this rigorous process, we gained valuable insights into the antimicrobial potency of the Thai basil extracts, paving the way for further exploration of their potential applications in various fields.

### 2.14. Antithrombotic Activity

To test the extracts’ ability to prevent blood clotting (antithrombotic activity), a modified version of a standard method called the prothrombin time (PT) test was applied [19]. First, a solution called “thromboplastin–calcium chloride” was prepared, which is a key component of the PT test. This solution was made using a special powder called “tissue thromboplastin” extracted from rabbit tissue (Sigma Aldrich, St. Louis, MO, USA). Importantly, this solution was then warmed up to body temperature (37 °C) for 30 min. This ensured that the solution was working at its best for the next step of the experiment, where it would be mixed with the extracts and blood samples to measure the clotting time.

The assay involved pipetting 100 µL of citrated (3.8% sodium citrate) rabbit plasma (National Laboratory Animal Center, Mahidol University, Thailand) prepared with a range of Thai basil extract concentrations (ranging from 100 to 500 µg/mL) into a 12 × 75 mm test tube. This mixture was preincubated for 1 min at 37 °C to allow the extract to interact with the clotting factors. Subsequently, 200 µL of the prewarmed PT reagent was added, and a stopwatch was immediately started. The time taken for a visible clot to form after mixing the plasma and reagent was recorded as the prothrombin time. Warfarin sodium (Sriprasit Pharma, Bangkok, Thailand), a known anticoagulant, was included as a positive control to validate the assay. This modified PT assay provided a quantitative measure of the extracts’ ability to prolong the blood clotting time, thereby indicating their potential antithrombotic activity. The comparison with a positive control ensured the reliability and accuracy of the results.

### 2.15. Statistical Analysis

Each measurement was repeated three times (triplicate) and averaged with its standard deviation to show typical values. Differences between averages were statistically tested using ANOVA and Duncan’s test (*p* < 0.05) in SPSS 16.0 (SPSS Inc., Chicago, IL USA).

## 3. Results

### 3.1. Proximate Analysis

The *O. basilicum* var. *thyrsiflorum* and Jumbo 4320 differed significantly in their main chemical compositions. Thyrsiflorum had higher moisture and carbohydrate content (39.73% ± 0.32% and 38.38% ± 0.30%, respectively), while Jumbo 4320 contained significantly more ash and protein (29.94% ± 0.16% and 27.31% ± 0.13%, respectively) (*p* < 0.05 for all comparisons).

Using three distinct solvents (water, ethanol, and ethyl acetate), this study investigated the yield percentages of crude extracts from two Thai basil varieties, *O. basilicum* var. *thyrsiflorum* and *O. basilicum* cv. Jumbo 4320. Notably, all solvent-mediated extractions produced statistically significant differences in yield (*p* < 0.05). Interestingly, thyrsiflorum consistently yielded more extract than Jumbo 4320, with water extraction exhibiting the highest overall yield at 15.12 ± 0.08%.

### 3.2. Phytochemical Profiling

#### 3.2.1. Chlorophylls and Carotenoid Profile of Thai Basil Extracts

Thai basil extracts prepared with ethanol and ethyl acetate, as analyzed by reverse-phase HPLC and UV–vis spectra, were found to contain chlorophyll *a*, chlorophyll *b*, pheophytin *a*, and pheophytin *b*, including lutein, while hot-water extracts lacked these pigments. Table 1 details the chlorophyll and lutein content of both *O. basilicum* var. *Thyrsiflorum* and *O. basilicum* cv. Jumbo 4320 extracts were obtained using different solvents. Notably, all chlorophyll and lutein levels differed significantly (*p* < 0.05) between ethanol and ethyl acetate extracts, with ethanol yielding higher chlorophyll b, pheophytin a and b, total chlorophylls, and lutein. Interestingly, ethyl acetate only surpassed ethanol in chlorophyll content. Additionally, var. *Thyrsiflorum* extracts consistently exhibited higher chlorophyll a, b, total, and lutein content than cv. Jumbo 4320 (*p* < 0.05), while cv. Jumbo 4320 extracts contained significantly more pheophytin a and b (*p* < 0.05). This reveals the influence of both solvent choice and basil variety on the extracted pigment profile.

Ethyl acetate proved most adept at extracting chlorophyll a from *O. basilicum* var. *Thyrsiflorum*, garnering 4188.29 ± 6.96 µg/g. However, ethanol shone for chlorophyll *b*, total chlorophylls, and lutein in the same variety, yielding 1355.73 ± 0.86 µg/g, 7557.84 ± 6.25 µg/g, and 405.24 ± 0.23 µg/g, respectively. Notably, for pheophytin *a* and *b*, *O. basilicum* cv. Jumbo 4320 favored ethanol extraction, achieving 4127.16 ± 1.95 µg/g and 124.58 ± 0.37 µg/g.

#### 3.2.2. Phenolic Profiling of Thai Basil Extracts

High-performance liquid chromatography (HPLC) analysis revealed a rich variety of phenolic acids and flavonoids in Thai basil extracts. Notably, gallic acid, catechin, apigenin, and several phenolic acids (including caffeic, coumaric, ferulic, and sinapic acids) were identified. See Table 2 for a detailed breakdown of the phenolic profiles for both *O. basilicum* var. *Thyrsiflorum* and *O. basilicum* cv. Jumbo 4320 crude extracts.

### 3.3. Total Phenolic and Flavonoid Contents

**Figure 1:**
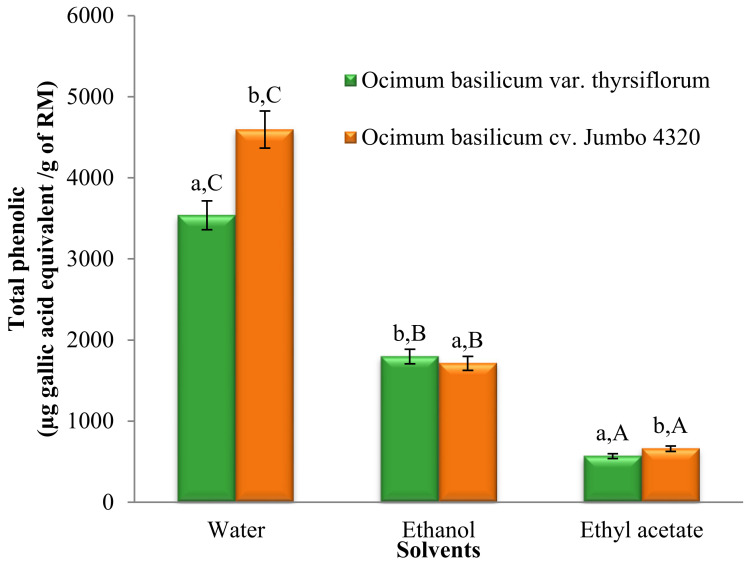
Total phenolic contents of Thai basil crude extracts. Data are mean ± SD (*n* = 3). Different letters over the error bars denote that the means differed significantly (*p* < 0.05) in species and solvents.

The Folin–Ciocalteu method was employed to quantify the total phenolic content of *O. basilicum* var. *thyrsiflorum* and *O. basilicum* cv. Jumbo 4320 extracts obtained using water, ethanol, and ethyl acetate. Figure 1 depicts the total phenolic content variations across all extracts.

Significant differences in the total phenolic content (TPC) were observed across all solvent extracts (*p* < 0.05). Water extracts exhibited the highest TPC, followed by ethanol and ethyl acetate extracts. Additionally, significant differences (*p* < 0.05) were found between the two basil varieties. Notably, *O. basilicum* cv. Jumbo 4320 extracted with water boasted the highest TPC, reaching 4596.19 ± 3.07 µg gallic acid equivalent/g of raw material (RM).

### 3.4. Total Flavonoid Contents

**Figure 2:**
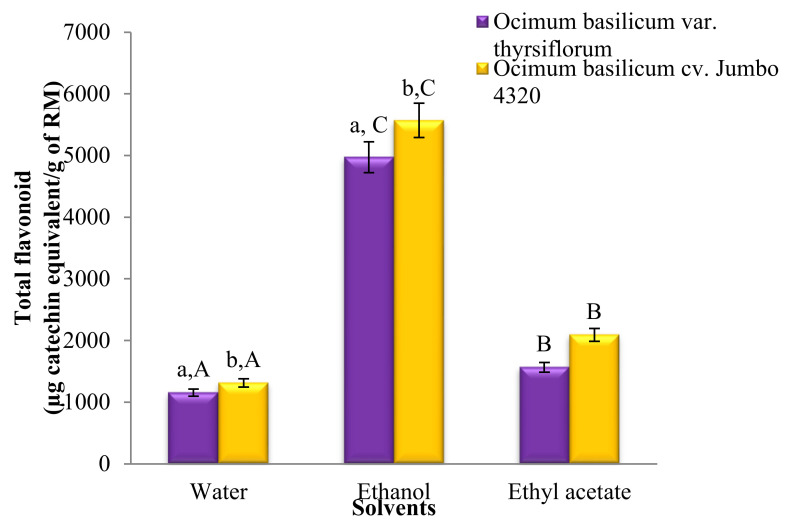
Total flavonoid contents of Thai basil crude extracts. Data are mean ± SD (*n* = 3). ^a,b,A,B,C^ Different letters over the error bars denote that the means differed significantly (*p* < 0.05) in species and solvents.

Figure 2 showcases the significantly different (*p* < 0.05) total flavonoid contents across all solvent extracts. Notably, ethanol extracts possessed the highest flavonoid levels, followed by ethyl acetate and water extracts. Interestingly, *O. basilicum* cv. Jumbo 4320 consistently exhibited higher flavonoid content than *O. basilicum* var. *thyrsiflorum*, with statistically significant differences (*p* < 0.05). However, the flavonoid content in ethyl acetate extracts for both species remained statistically comparable (*p* > 0.05).

Figure 2 reveals significant differences (*p* < 0.05) in the total flavonoid content across all solvent extracts. Notably, ethanol reigned supreme, followed by ethyl acetate and water extracts. Interestingly, both water and ethanol extracts of *O. basilicum* cv. Jumbo 4320 boasted significantly higher flavonoid levels than *O. basilicum* var. *thyrsiflorum* (*p* < 0.05). However, the flavonoid content remained statistically comparable for both species when extracted with ethyl acetate (*p* > 0.05). The greatest victor, *O. basilicum* cv. Jumbo 4320 extracted with ethanol, achieved a phenomenal flavonoid content of 557.12 ± 14.27 mg quercetin equivalent/100 g raw material.

### 3.5. Antioxidant Activity

#### 3.5.1. DPPH Radical Scavenging Activity

All extracts displayed dose-dependent DPPH radical scavenging activity. BHT and ascorbic acid, used as positive controls, confirmed the assay’s effectiveness. Table 3 reports the calculated IC50 values for 15 min of incubation. Interestingly, both solvent and basil species significantly influenced the antioxidant activity (*p* < 0.05). Water and ethanol extracts revealed a significant difference in activity between *O. basilicum* var. *thyrsiflorum* and cv. Jumbo 4320; no such difference was observed for the ethyl acetate extracts. Notably, *O. basilicum* cv. Jumbo 4320 extracted with water emerged as the most potent antioxidant, boasting an IC50 value of 48.52 ± 1.15 mg/mL.

#### 3.5.2. Ferric-Reducing Antioxidant Power Assay

The FRAP analysis revealed significant differences (*p* < 0.05) in antioxidant activity across the solvent extracts, with ethanol reigning supreme, followed by water and ethyl acetate extracts. Similarly, a significant difference (*p* < 0.05) was observed between the two basil species. According to Wong et al.’s classification [20], both ethanol and water extracts of *O. basilicum* var. *thyrsiflorum* and the ethanol extract of *O. basilicum* cv. Jumbo 4320 fell within the “medium” range (10–100 µmol Fe(II)/g) of antioxidant activity. All other extracts exhibited “low” activity (<10 µmol Fe(II)/g). Notably, *O. basilicum* var. *thyrsiflorum* extracted with ethanol claimed the top spot, with a FRAP value of 0.0186 ± 0.00 mmol Fe^2+^/g.

### 3.6. Cytotoxicity of Extracts

The cytotoxicity of the Thai basil (*O. basilicum*) extracts toward HepG2 cells was assessed using the MTT assay. Increasing concentrations (50–500 μg/mL) of both *O. basilicum* var. *thyrsiflorum* and *O. basilicum* cv. Jumbo 4320 extracts progressively reduced cell survival in a dose-dependent manner. This effect likely involves the inhibition of a mitochondrial enzyme by the extracts, ultimately affecting cell viability. Table 4 presents the lethal concentration (LC_50_) values for cytotoxicity of Thai basil’s extracts. Cell viability assays revealed remarkable nontoxic activity for all Thai basil crude extracts. Notably, the LC50 values against the tested cell lines consistently exceeded 200 μg/mL, suggesting a significant margin of safety.

### 3.7. Cellular Antioxidant Activity (CAA) by DCFH-DA Assay

**Figure 3:**
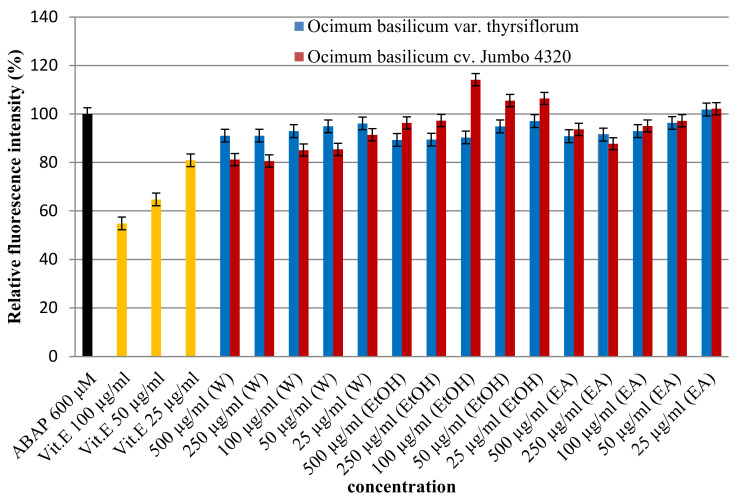
Cellular antioxidant activities of Thai basil crude extracts by the DCFH-DA assay.

The cell-based assay was applied to assess the antioxidant activity of Thai basil extracts (*O. basilicum*) against oxidative stress in HepG2 cells. Cells were treated with various extract concentrations (25–500 µg/mL) and challenged with a free radical inducer (ABAP). As shown in Figure 3, dose-dependent increases in Thai basil effectively countered ABAP-induced oxidative damage. Interestingly, significant differences (*p* < 0.05) were observed in antioxidant activity based on both the solvent used (water, ethanol, and ethyl acetate) and basil variety (*O. basilicum* var. *thyrsiflorum*, cv. Jumbo 4320). Notably, *O. basilicum* cv. Jumbo 4320 extracted with water emerged as the most potent antioxidant, exhibiting the highest cellular protection (80.62 ± 0.00% relative fluorescence intensity) against ABAP-induced free radical damage. This suggests that Thai basil, particularly cv. Jumbo 4320 extracted with water, possesses strong cellular radical scavenging ability.

### 3.8. Antimicrobial Activity by Disk Diffusion Method

When tested against various pathogenic strains, the antimicrobial activity of Thai basil (*O. basilicum*) extracts was generally weak, except for specific instances. While most bacteria remained unaffected by all water, ethanol, and ethyl acetate extracts, Bacillus cereus showed vulnerability to both the ethyl acetate extract of *O. basilicum* var. *thyrsiflorum* and the water extract of *O. basilicum* cv. Jumbo 4320. Interestingly, the observed antimicrobial potency remained lower than the reference standard, chloramphenicol. This aligns with previous findings by Shafique et al. [21], who documented the minimal-to-no activity of sweet basil ethanolic extracts against common bacterial strains (Table 5).

### 3.9. Antithrombotic Activity by Prothrombin Time Assay

Thai basil extracts demonstrated dose-dependent antithrombotic activity, prolonging the blood clotting time (prothrombin time) as their concentration increased. This effect was measured using the prothrombin time assay, with data presented in Table 6. Interestingly, the *O. basilicum* var. *thyrsiflorum* water extract at 100 µg/mL proved significantly more potent than its counterpart from cv. Jumbo 4320. While differences in the solvent used (water, ethanol, and ethyl acetate) significantly impacted the antithrombotic activity at 100 µg/mL, this did not hold for higher water and ethanol extract concentrations. Notably, ethyl acetate extracts at 500 µg/mL exhibited the highest prothrombin time prolongation, with both varieties reaching around 77 s. However, the overall potency remained lower than the reference standard, warfarin sodium.

## 4. Discussion

Our results align with previous reports on solvent effectiveness for extracting specific pigments from *O. basilicum* and other leafy greens. Notably, ethanol’s superior extraction of chlorophyll b, total chlorophylls, and lutein from *O. basilicum* var. *Thyrsiflorum* agrees with observations by Rajkumar et al. [22], who attributed this to ethanol’s ability to disrupt chloroplast membranes and enhance pigment release. In contrast, ethyl acetate’s higher affinity for chlorophyll in both varieties’ echoes Benković et al. [23], who suggested its polarity favors extracting this specific chlorophyll from plant tissues. These findings solidify the importance of solvent selection for optimizing the extraction of desired pigments based on their solubilities and interactions with different solvents.

The consistent superiority of *O. basilicum* var. *Thyrsiflorum* over cv. Jumbo 4320 in chlorophyll a, b, total, and lutein content aligns with observations by Kang et al. [24]. They attributed this difference to variations in genetic makeup and metabolic pathways between cultivars, leading to differential pigment biosynthesis and accumulation. Our findings further substantiate the influence of genotype on final pigment profiles in Thai basil extracts.

Interestingly, *O. basilicum* cv. Jumbo 4320 favored ethanol extraction for pheophytin a and b, potentially indicating distinct pigment degradation patterns in this variety. Pheophytins are chlorophyll derivatives formed by the enzymatic removal of magnesium, and their presence can influence the color and stability of extracts. Further research is needed to elucidate the factors behind the observed cv. Jumbo 4320’s preference for ethanol in extracting these degradation products.

Overall, the present study expands our understanding of the complex interplay between solvent, basil variety, and the resulting pigment profile in Thai basil extracts. These findings hold potential for optimizing extraction protocols to target specific pigments for applications in food science, nutraceuticals, and natural colorants.

Our findings align with previous reports on solvent-dependent variations in the antioxidant activity of plant extracts. Notably, the higher DPPH scavenging activity observed in water and ethanol extracts of *O. basilicum* var. *thyrsiflorum* compared to ethyl acetate extracts echoes with the observations of Zhang et al. [25]. They attributed this discrepancy to the differential extraction of specific phenolic and flavonoid compounds, depending on the solvent’s polarity and ability to solubilize diverse antioxidant moieties. This suggests that water and ethanol extract a broader spectrum of antioxidants from this basil variety, contributing to their enhanced activity.

The significantly higher antioxidant activity exhibited by *O. basilicum* cv. Jumbo 4320 extracted with water compared to *O. basilicum* cv. *Thyrsiflorum* aligns with the findings of [26]. They attributed similar observations to variations in the type and concentration of antioxidant compounds present in different cultivars. Our results strengthen the notion that genotype plays a crucial role in determining the overall antioxidant potency of Thai basil extracts.

Interestingly, in contrast to water and ethanol extracts, no significant difference in activity was observed between the two basil varieties for ethyl acetate extracts. This suggests that ethyl acetate might preferentially extract a specific set of antioxidants present in both varieties, resulting in comparable DPPH scavenging potential. Further investigation regarding the specific antioxidant compounds extracted by ethyl acetate would be insightful in elucidating this observation.

Overall, this study highlights the complex interplay between solvent choice, basil variety, and the resulting antioxidant activity in Thai basil extracts. These findings hold potential for optimizing extraction protocols to maximize the recovery of potent antioxidants with potential applications in the food, pharmaceutical, and nutraceutical industries.

The isolation and purification of chlorophyll from spinach leaves was obtained through a modified method. Seven phenolic compounds, gallic acid, catechin, apigenin, caffeic acid, coumaric acid, ferulic acid, and sinapic acid, were identified in both Thai basil varieties through chromatography with standards. Gallic acid, the most abundant phenolic in all extracts, showed higher levels in *O. basilicum* var. *Thyrsiflorum* than in cv. Jumbo 4320, regardless of the solvent used. Catechin displayed variable presence, detected only in water and ethanol extracts of Thyrsiflorum and in the water extract of cv. Jumbo 4320. Apigenin was unique to *O. basilicum* cv. Jumbo 4320′s water extract. Caffeic acid and coumaric acid were predominantly found in water and ethanol extracts, with higher levels in *O. basilicum* cv. Jumbo 4320 for caffeic acid and comparable levels for coumaric acid between varieties. Notably, both were absent in ethyl acetate extracts. Ferulic acid remained undetectable, while sinapic acid was present in ethanol extracts of both varieties and in the water extract of cv. Jumbo 4320. The total phenolic contents of both varieties of extracts align with reports that more polar solvents, like aqueous methanol/ethanol, often yield higher phenolic recoveries [27]. Among all samples, *O. basilicum* cv. Jumbo 4320 extracted with ethanol yielded the highest total flavonoid content, reaching 5571.16 ± 14.27 µg catechin equivalent/g of raw material (RM). This agrees with studies suggesting preferential extraction of flavonoids by moderately polar solvents like ethanol [28].

While numerous reports, like Grayer et al. [29], identify specific phenolic and flavonoid compounds in *O. basilicum*, like cinnamic acid, the exact chemical composition of sweet basil is highly variable. It can be influenced by factors like the cultivar, season, environmental conditions, genetic makeup, and even where the plant was grown [30]. To capture this diverse range of bioactive compounds, researchers often turn to polar solvents, particularly aqueous mixtures of ethanol, methanol, acetone, and ethyl acetate. Among these, ethanol stands out for its efficiency in extracting polyphenols while remaining safe for consumption [27].

Several studies, including Chaisawadi et al. [31], have consistently reported limited or no antimicrobial activity of sweet basil extracts against common bacterial strains, like *Bacillus cereus*, *Staphylococcus aureus*, *Bacillus subtilis*, *Escherichia coli*, *Enterobacter aerogenes*, and *Pseudomonas aeruginosa*.

Blood clotting involves a complex interplay of mechanisms, ultimately driven by the enzyme thrombin. This final player in a cascading series of reactions transforms soluble fibrinogen into insoluble fibrin, solidifying the blood to prevent excessive bleeding. Platelets trigger this process by activating prothrombin into thrombin [32].

While the prothrombin time (PT) test does not perfectly mirror real-life blood clotting, it remains a valuable tool in the lab for assessing the function of specific coagulation factors. PT measures the activity of several factors in the extrinsic and common pathways, particularly those requiring vitamin K for their synthesis (factors II, VII, and X). This sensitivity makes PT a reliable indicator of how efficiently the “tissue factor” pathway (triggered by injury) functions. However, its reliance on isolated factors in a test tube setting means it may not always capture the complex interplay of factors during actual bleeding events.

## 5. Conclusions

This study delved into the chemical composition and bioactivities of two Thai basil varieties (*Ocimum basilicum* var. *thyrsiflorum* and *Ocimum basilicum* cv. Jumbo 4320) extracted with water, ethanol, and ethyl acetate. Notably, water extraction from Jumbo 4320 maximized its phenolic content and antioxidant activity (DPPH and DCFH-DA), suggesting its potential for healthy food applications. Conversely, ethanol extraction excelled in extracting flavonoids and resulted in the strongest FRAP antioxidant activity, making it ideal for obtaining flavonoid-rich ingredients. Interestingly, both varieties’ ethyl acetate extracts displayed the highest prothrombin time extension, indicating their potential for anticoagulant effects. While most extracts lacked significant antimicrobial activity, all exhibited low cytotoxicity, ensuring their relative safety for consumption. Overall, this study highlights the potential of both Thai basil varieties as sources of functional food ingredients with diverse bioactivities, depending on the extraction solvent. Further research should explore specific applications and optimize the extraction processes for desired bioactivities, addressing limitations like limited evaluation against other foodborne pathogens or the lack of mechanism exploration. Additionally, future directions could involve in vivo studies or specific product development using optimized extracts.

## Figures and Tables

**Table 1:** Phytochemical profile of Thai basil crude extracts.

Phytochemical Contents (µg/g RM)	*O. basilicum* var. *thyrsiflorum*			*O. basilicum* cv. Jumbo 4320		
	Solvent			Solvent		
	Water	Ethanol	Ethyl Acetate	Water	Ethanol	Ethyl Acetate
Chlorophyll *a*	ND	2191.18 ± 3.61 ^b,e^	4188.29 ± 6.96 ^d,f^	ND	1547.30 ± 2.69 ^a,e^	3369.72 ± 6.58 ^c,f^
Chlorophyll *b*	ND	1355.73 ± 0.86 ^b,f^	1280.29 ± 0.79 ^d,e^	ND	1130.35 ± 0.10 ^a,f^	1089.77 ± 1.92 ^c,e^
Pheophytin *a*	ND	3916.84 ± 1.06 ^a,f^	1159.26 ± 2.75 ^c,e^	ND	4127.16 ± 1.95 ^b,f^	1707.61 ± 1.87 ^d,e^
Pheophytin *b*	ND	94.09 ± 0.72 ^a,f^	20.34 ± 0.61 ^c,e^	ND	124.58 ± 0.37 ^b,f^	70.47 ± 2.36 ^d,e^
Lutein	ND	405.24 ± 0.23 ^b,f^	374.31 ± 0.62 ^d,e^	ND	360.51 ± 0.30 ^a,f^	306.01 ± 0.68 ^c,e^
Total chlorophylls	ND	7557.84 ± 6.25 ^b,f^	6648.18 ± 11.11 ^d,e^	ND	6929.39 ± 5.11 ^a,f^	6237.57 ± 12.73 ^c,e^

Note: Each value is mean ± SD (*n* = 3); ND means not detected. ^a–f^ Data within the same row with different superscripts are significantly different (*p* < 0.05).

**Table 2:** Phenolic profiles of Thai basil crude extracts.

Phenolic Contents (µg/g RM)	*O. basilicum* var. *thyrsiflorum*			*O. basilicum* cv. Jumbo 4320		
	Solvent			Solvent		
	Water	Ethanol	Ethyl Acetate	Water	Ethanol	Ethyl Acetate
Gallic acid	248.60 ± 7.61 ^b,f^	186.98 ± 0.66 ^c,e^	189.55 ± 1.14 ^e^	227.70 ± 2.39 ^a,g^	195.78 ± 0.93 ^d,f^	190.14 ± 0.56 ^e^
Catechin	111.49 ± 7.65 ^a,e^	131.94 ± 5.10 ^f^	ND	201.05 ± 9.18 ^b^	ND	ND
Apigenin	ND	ND	ND	29.56 ± 0.65	ND	ND
Caffeic acid	19.52 ± 0.71 ^a,e^	32.83 ± 2.32 ^f^	ND	63.76 ± 0.79 ^b,f^	32.94 ± 2.50 ^e^	ND
Coumaric acid	178.81 ± 0.54 ^f^	175.00 ± 0.55 ^c,e^	ND	178.65 ± 0.36 ^e^	188.69 ± 2.14 ^d,f^	ND
Ferulic acid	ND	ND	ND	ND	ND	ND
Sinapic acid	ND	107.53 ± 0.96 ^c^	ND	104.88 ± 1.08 ^e^	111.42 ± 1.01 ^d,f^	ND

Note: Each value is mean ± SD (*n* = 3); ND means not detected. ^a–g^ Data within the same row with different superscripts are significantly different (*p* < 0.05).

**Table 3:** Antioxidant activities of Thai basil crude extracts in the DPPH assay and FRAP assay.

Solvents	DPPH IC_50_ (mg/mL)		FRAP (mmol Fe^2+^/g)	
	*O*. *basilicum* var. *thyrsiflorum*	*O*. *basilicum* cv. Jumbo 4320	*O*. *basilicum* var. *thyrsiflorum*	*O*. *basilicum* cv. Jumbo 4320
Water	105.62 ± 3.77 ^b,e^	48.52 ± 1.15 ^a,c^	0.0139 ± 0.00 ^b,d^	0.0090 ± 0.00 ^a,d^
Ethanol	53.88 ± 0.74 ^a,c^	60.54 ± 0.52 ^b,d^	0.0186 ± 0.00 ^b,e^	0.0153 ± 0.00 ^a,e^
Ethyl acetate	72.48 ± 5.57 ^ns,d^	82.09 ± 3.18 ^ns,e^	0.0012 ± 0.00 ^a,c^	0.0016 ± 0.00 ^b,c^
BHT	0.18 ± 0.00	0.18 ± 0.00	2.6159 ± 0.02	2.6159 ± 0.02
Ascorbic acid	0.06 ± 0.00	0.06 ± 0.00		

Note: Each value is mean ± SD. ^a–e^ Data within the same row with different superscripts are significantly different (*p* < 0.05). ^ns^ Not significant.

**Table 4:** Cytotoxicity of Thai basil crude extracts.

Solvents	Cytotoxicity LC_50_ (µg/mL)	
	*O*. *basilicum* var. *thyrsiflorum*	*O*. *basilicum* cv. Jumbo 4320
Water	966.50 ± 39.07 ^b,B^	567.49 ± 4.17 ^a,A^
Ethanol	688.85 ± 31.37 ^A^	632.19 ± 20.92 ^B^
Ethyl acetate	624.36 ± 30.54 ^A^	640.82 ± 11.96 ^B^

Note: Each value is mean ± SD (*n* = 5). ^a,b,A,B^ Data within the same row with different superscripts are significantly different (*p* < 0.05) in species and solvents.

**Table 5:** Antimicrobial activity of Thai basil crude extracts.

Type Strains	Inhibition Zone (mm)						
	*O. basilicum* var. *thyrsiflorum* (800 mg RM/mL)			*O. basilicum* cv. Jumbo 4320 (800 mg RM/mL)			Chloramphenicol (16.67 mg/mL)
	Water	Ethanol	Ethyl Acetate	Water	Ethanol	Ethyl Acetate	
*Bacillus cereus*	-	-	7.00 ± 0.00	6.5 ± 0.00	-	-	30.4 ± 1.33 ^b^
*Bacillus subtilis*	-	-	-	-	-	-	13.3 ± 0.98 ^a^
*Enterobacter aerogenes*	-	-	-	-	-	-	32.0 ± 0.99 ^c^
*Escherichia coli*	-	-	-	-	-	-	12.6 ± 1.03 ^a^
*Pseudomonas aeruginosa*	-	-	-	-	-	-	32.1 ± 1.03 ^c^
*Staphylococcus aureus*	-	-	-	-	-	-	31.5 ± 0.00 ^c^

Note: Each value is mean ± SD (*n* = 3). - No inhibition zone. ^a–c^ Data within the same column with different superscripts are significantly different (*p* < 0.05).

**Table 6:** Prothrombin time of Thai basil crude extracts on rabbit plasma.

Solvents	Prothrombin Time (Second)					
	*Ocimum basilicum* var. *thyrsiflorum* (µg RM/mL)			*Ocimum basilicum* cv. Jumbo 4320 (µg RM/mL)		
	100	250	500	100	250	500
Water	34.7 ± 0.58 ^b,A^	43.0 ± 0.00 ^B^	45.3 ± 0.58 ^AB^	31.0 ± 1.00 ^a,B^	42.0 ± 1.00 ^B^	46.0 ± 1.00 ^B^
Ethanol	38.7 ± 1.15 ^B^	47.7 ± 0.58 ^C^	53.7 ± 3.21 ^B^	37.0 ± 1.00 ^C^	43.7 ± 4.93 ^B^	46.3 ± 0.58 ^B^
Ethyl acetate	57.3 ± 2.52 ^C^	69.3 ± 4.04 ^D^	78.3 ± 17.56 ^C^	54.3 ± 1.15 ^D^	69.7 ± 1.53 ^C^	77.3 ± 3.21 ^C^
Warfarin (6 mg/mL)	144.3 ± 0.58 ^D^	144.3 ± 0.58 ^E^	144.3 ± 0.58 ^D^	144.3 ± 0.58 ^E^	144.3 ± 0.58 ^D^	144.3 ± 0.58 ^D^
Control	33.3 ± 1.53 ^A^	33.3 ± 1.53 ^A^	33.3 ± 1.53 ^A^	33.3 ± 1.53 ^A^	33.3 ± 1.53 ^A^	33.3 ± 1.53 ^A^

Note: Each value is mean ± SD (*n* = 4). ^A–E^ Data within the same row with different superscripts are significantly different (*p* < 0.05) for different species within the same concentration of sample.

## Data Availability

The original contributions presented in the study are included in the article, further inquiries can be directed to the corresponding author.
